# Low-dose daylight exposure induces nitric oxide release and maintains cell viability in vitro

**DOI:** 10.1038/s41598-023-43653-2

**Published:** 2023-09-28

**Authors:** Gareth Hazell, Marina Khazova, Paul O’Mahoney

**Affiliations:** UK Health Security Agency, Chilton, Didcot, OX11 0RQ UK

**Keywords:** DNA damage and repair, Cell biology, Molecular biology, Environmental sciences, Biomarkers

## Abstract

Any potential positive effects conferred via sunlight exposure have to be carefully balanced against carcinogenic effects. Here we provide evidence UK sunlight exposure upregulates the cardio protectant nitric oxide (NO) within in vitro skin cell lines with negligible increases in DNA damage and cell death at 1 SED, when compared against unexposed samples. The whole of the ultraviolet A (UV-A) spectrum appears to be responsible for NO release, with efficiency higher at exposures closer to shorter UV-A wavelengths and decreasing with wavelength increases. These results support further in vivo work, which could be of benefit for demographics such as the elderly (that exhibit a natural decline in NO bioavailability).

## Introduction

Over-exposure to sunlight increases risk of skin cancers, erythema, cataracts, premature skin aging and a weakened immune system^1–3^. However, human life evolved under the sun and insufficient exposure also harbours adverse health consequences. Vitamin D circulation promoting healthy bones and prevention of rickets, alongside the blood pressure lowering effect of UV-A are the most well documented examples of this^4,5^. However, recent findings suggest that sunlight may have a far greater systemic effect reducing risk of some cancers, regulating hormones (such as melatonin and serotonin for cognitive function), preventing childhood myopia, (recently recognised by the World Health Organisation as an emerging health risk), modulating hormones that directly affect the neuroendocrine axis (for example beta endorphin and acetylcholine) and preventing metabolic disorders^6–11^. Hence, while sunlight only interacts directly with the skin and eyes, it derives systemic effects via intermediates that may, in turn, be much more far reaching and important for many physiological functions^7,9,10^.

The UV-A and UV-B wavelengths are largely responsible for this phenomenon. Shorter UV-B (280–320 nm) is predominantly responsible for skin carcinogenesis, directly interacting with thymine and cytosine bases within DNA deriving ‘signature’ mutations termed photolesions on contact^12^. Conversely, UV-A (320–400 nm) does not cause significant direct DNA damage, instead interacting with different cellular components^12^ such as salts situated in the skin, liberating these latter mentioned compounds as nitric oxide (NO)^13–15^. In turn, NO potentially drives many physiological processes systemically (such as cardio protection) in vivo^5,16,17^. In this study we examine the effect of UK sunlight exposures up to 5 standard erythema doses (SED) on NO induction, direct DNA damage and cell survival. Our work is unique in that we have chosen to use actual UK sunlight, with dose rate typical for the UK summer months and not an artificial solar simulator to carry out many in-vitro exposures. Artificial light sources (such as xenon arc lamps) are useful for evaluation of the effects of sunlight in biological models as they streamline experiments subverting complications and uncertainties caused by weather and challenges of accurate exposure however, their use is not optimal for a complete assessment of real-life sunlight exposure as it is highly unlikely that the emission of the solar simulator is fully identical to natural sunlight, both spectrally and in terms of dose rate. Such data would provide useful input to the risk–benefit analysis of sunlight exposures for different demographic groups.

## Materials and methods

### Cell culture

Ten foreskin tissue resections (obtained from white skinned neonates approximately 7–10 days old) were placed in ‘transport medium’ derived from DMEM supplemented with 10% FBS (Gibco, 10500064), penicillin, streptomycin, amphotericin and gentamycin were also placed in the container and the samples shipped to site. Prior to sample receipt, informed consent was obtained from the child’s parent or legal guardian, and ethical approval put in place under the South-central Berkshire B ethics committee REF 22/SC/0411, IRAS ID 318321.

Cells were isolated from samples as described previously by Holliman et al.^13^. To summarise, tissue was cut into oblongs approximately 5–7 mm each side and digested overnight at 4 °C with 0.5 mg/ml Liberase (ROCHE 5401054001) diluted in 4.5 ml CnT-07 keratinocyte medium (CellnTech). Following digestion, the epidermal layer was removed with tweezers and mechanically dissociated in trypsin EDTA to form a single-cell keratinocyte (FSK) suspension upon pelleting. FSK were subsequently grown in CnT-07 medium, containing penicillin, streptomycin, amphotericin and gentamycin for 7 days—with removal of antibiotics and anti-fungal thereafter. The remaining dermis was used to isolate Fibroblasts (FSF) via placing an individual explant oblong into DMEM supplemented with 10% FBS penicillin, streptomycin, amphotericin and gentamycin for seven days, and endothelial cells (FSEC) by placing the remaining dermal explants into 2.5 mg/ml collagenase in HBSS (with Ca and Mg) at 37 °C for 1 h followed by CD31 magnetic Dynabead positive selection (Life Technologies, 11155D). Endothelial cells were then seeded within a gelatine-coated flask in Endothelial Cell Growth Medium MV (PromoCell, C-22020) containing penicillin, streptomycin, amphotericin and gentamycin, with both dermal cell lines having antibiotics and anti-fungal removed after 7 days. During growth to confluency all cell lines were maintained in a 37 °C, 5% CO_2_ humidified environment. Cells used for experiments were low passage and non-immortalised (passage 1–5), to negate adverse effects visualised in terms of morphological, genetic and proliferative changes visualised when high passage immortalised cells are opted for.

### UK sunlight exposure of cell culture samples

Experiments in this study used exposure to actual UK sunlight on the roof of the UK Health Security Agency (UKHSA) in Chilton (51.575°, − 1.318°) from May to August 2021 under rain-free conditions, mostly on days with low cloud cover. Real-time erythema and UV-A irradiance data from a co-located UKHSA solar monitoring ground station (https://uk-air.defra.gov.uk/data/uv-index-graphs) was used to control the duration of exposure to reach the required dose. Spectral irradiance was collected by UKHSA reference double-grating DTMc300 spectroradiometer (Bentham Instruments, Reading, UK) at the same location. The Solar monitoring ground station comprises SL-501A-UV Robertson-Berger meter (SolarLight Inc, Glenside, USA) and SD-104Acos (Macam Photometrics Ltd, Livingstone, UK) sensors for erythema effective irradiance and UV-A irradiance measurements with the sampling rate of 1.2 s.

**Figure 1:**
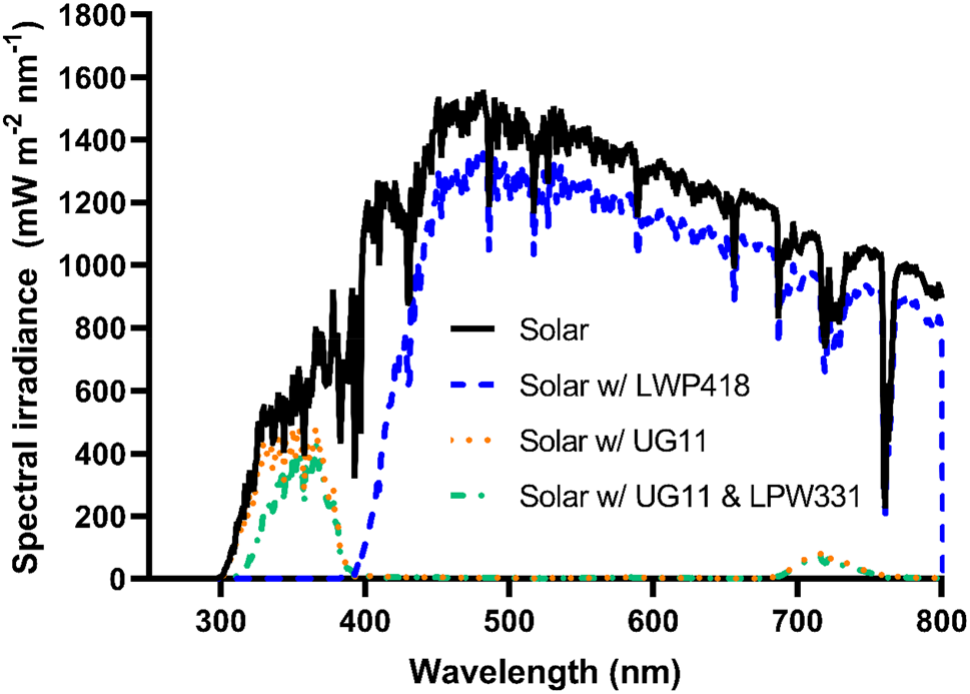
Spectral irradiance of sunlight at midday in Chilton, and corresponding filtered sunlight.

UG11 solar-blind, LPW331 and LPW418 long pass filters shown in Fig. 1 were used to select UV and visible/infra-red spectral ranges, respectively; exposure to the full solar spectrum was carried out through a fused silica blank. Exposures were adjusted for filter transmittance and nominally identical exposures within each set were achieved by controlling exposure duration using real-time irradiances from the solar monitoring ground station. Full solar spectrum, UV only (< ~ 400 nm) and visible/IR only (> ~ 420 nm) samples were compared against an unexposed control shielded from light. To subvert the effects of heat shown within preliminary work to upregulate assessed markers of nitric oxide and DNA damage a purpose-built temperature-controlled stage was used. The stage provided constant temperature of the cell culture within ± 1 °C tolerance regardless of external conditions by cooling or heating samples.

Exposure doses were grouped as low (1–2 SED), medium (3–4 SED) and high (≥ 5 SED) to irradiate in vitro primary skin cell types isolated from tissue biopsies as highlighted above; quoted doses are expressed as a dose equivalent of full spectrum irradiation.

### Exposure of cell culture samples by ‘Sol-2’ solar simulator

**Figure 2:**
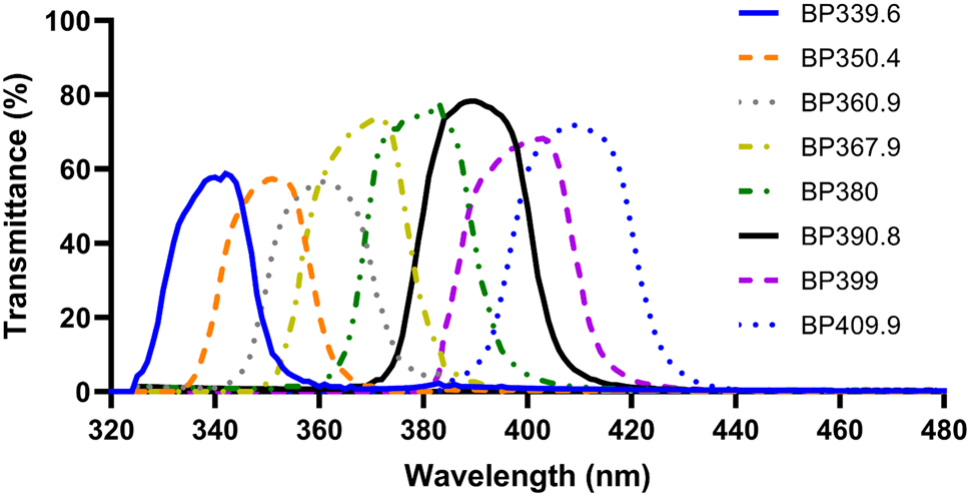
Transmittance of filters used to evaluate UV-A spectral response of nitric oxide release.

The SOL-2 solar simulator (Dr. Hönle AG UV—Technologie) with a range of bandpass filters shown in Fig. 2 was used for detailed investigation of cell survival and spectral response of NO release. Similar to sunlight exposures, duration of irradiation was adjusted to account for spectral power distribution of illumination source and filter transmission to provide identical exposure doses of 1.9 J/cm^2^. In addition to unexposed control, heat control samples were used quantifying heat-related contributions caused by filter absorption.

### Nitric oxide detection in cell lines using DAF-FM DA and DAX-J2 red

DAF-FM diacetate (DAF-FM DA) and DAX-J2 RED (DAX J2) are highly specific cell permeable dyes, that unlike other quantitative methods for NO release assess NO directly, and not via by-products such as nitrite or enzymatic NO synthase (NOS). This allows only NO generated within the cell itself to be detected.

Loading of either DAF-FM DA (Molecular Probes, D23844) or DAX J2 (AAT Bio, 16301-AATB) was performed as described previously^13^. To summarise, the compounds were used separately and were added at a 5 mM concentration in DMSO to 1 ml of culture media devoid of Phenol-red and foetal bovine serum. After addition, the compounds were incubated for 45 min at 37 °C prior to irradiation. Media containing either DAX-J2, or DAF FM DA were then removed, and the cells washed with HBSS^−/−^ (SIGMA, H6648). After washing the cells were removed from the plate by Trypsin–EDTA solution. Cells were then centrifuged and re-suspended in Dulbecco PBS^+/+^ (SIGMA, D8662). Media without phenol red and FBS were used as previous work has inferred that the compounds react poorly in the presence of phenol red and other serum-based components.

After the exposure 150 µl of the cell suspensions were then loaded in triplicate into 96-well plates. The suspensions then had 150 µl PBS with either 50 µg/ml propidium iodide (PI) (SIGMA, P4170), or SYTOX green (Thermo, R37168) added and were read with a Guava EasyCyte HT flow cytometer (Merk Millipore). Cells positive for PI or SYTOX green were excluded from further analysis. This technique was used for exposures using the SOL-2 solar simulator and actual UK sunlight.

### Western blotting

FSK were grown to between 95 and 100% confluency in 6 cm cell culture dishes in CNT-07 media. Media was then removed, and the cells rinsed with HBSS^−/−^. 3 ml of pre-warmed PBS^+/+^ was added to the cells and the cells were exposed to sunlight. Following exposure plates were returned to the incubator, washed in HBSS^−/−^ and then placed into fresh media. Cells were harvested at 2 h post-exposure, as previous data suggest that gamma H2AX used to quantify DNA damage is maximally upregulated at this timepoint^13,18^ Cells were harvested and western blots run via a standard protocol on 12% separating Tris–glycine gels with a 5% stacking buffer as described previously^18^. H2AX assessment was carried out against control antibody GAPDH (1:5000) (Santa-Cruz, SC25778) to ensure correct loading. Primary antibodies were detected via chemiluminescence with donkey anti-rabbit antibody (Santa-Cruz, SC2313) at a 1:10,000 concentration following incubation for 1 h at room temperature.

### Cell survival assays

Assessment of cell survival after light exposure was performed using a cell counting kit 8 (WST-8, Abcam ab228554). The kit assesses cell survival via the action of a tetrazolium salt that when ingested by live cells is emitted as a water-soluble formazan dye, staining surrounding media orange. The degree of colour change by measuring absorbance at 460 nm is then compared with a negative control to determine cell viability. Assays were performed at 24 h and 48 h post-exposure from 3 donors by addition of the cell counting kit at a 1 in 40 dilution in PBS^+/+^ to live cells. FSEC and FSK primary cell lines were chosen as these cells (unlike FSF) are grown as a monolayer as they are contact inhibited at confluency, hence cell numbers between wells would be approximately identical. FSK were grown in CNT-07 media and FSEC were grown in ECMV media, with light exposure being carried out when the cells reach 100% confluency. Alterations in cell survival at these timepoints were highlighted by carrying out the assay in triplicate, three times for each cell type and timepoint.

## Results

### UV-B mediated damage within samples is negligible with ‘low-dose’ (1 SED) UK sunlight

Any potential positive systemic effect of UK sunlight exposure has to be carefully balanced against damage that could occur in the skin, largely through UV-B exposure. Assessment of cell survival up to 48 h after full spectrum simulated sunlight exposure suggested that cells lying within both the epidermis (FSK) and dermis (FSEC) have a good tolerance to sunlight at 1 SED, comparable to unexposed samples. However, higher exposures reduced cell survival in both cell types, with endothelial cells having a lower tolerance to sunlight overall in these instances after 48 h. Experiments repeated on FSK with UV-B removed (using a LPW331 filter) and retaining UV-A only at 3 SED highlighted the fact that UV-B radiation in sunlight is the primary driver for cell death, reducing cell death by up to 90 percent.

We next assessed levels of direct DNA damage via H2AX expression, (a DNA repair enzyme and marker for assessment of double stranded DNA breaks). For this, full spectrum sunlight exposure was assessed against exposure to visible/infrared light and UV light only using UG11 and LPW filters (Fig. 4). FSKs were selected for these experiments as the cell forms 90% of the skin’s upper dermis, and as cancers with highest incidence rate linked to sunlight exposure originate in this cell line^19^. Evaluation of results highlighted that UV radiation conferred all direct DNA damage on FSK and was directly proportional to dose applied. Interestingly, when the UV spectra was isolated and used alone, it appeared to confer a trend towards greater H2AX upregulation than full spectrum irradiance for the equivalent erythema dose. We also noted that the H2AX upregulation in samples irradiated by the lowest dose of 1 SED of full spectrum sunlight was comparable to unexposed controls. This re-iterated the conclusion that low dose exposures to full spectrum UK sunlight confers insignificant adverse effects to skin cells in vitro.

### Low dose sunlight at 1 SED induces nitric oxide readily in all skin cells

**Figure 3:**
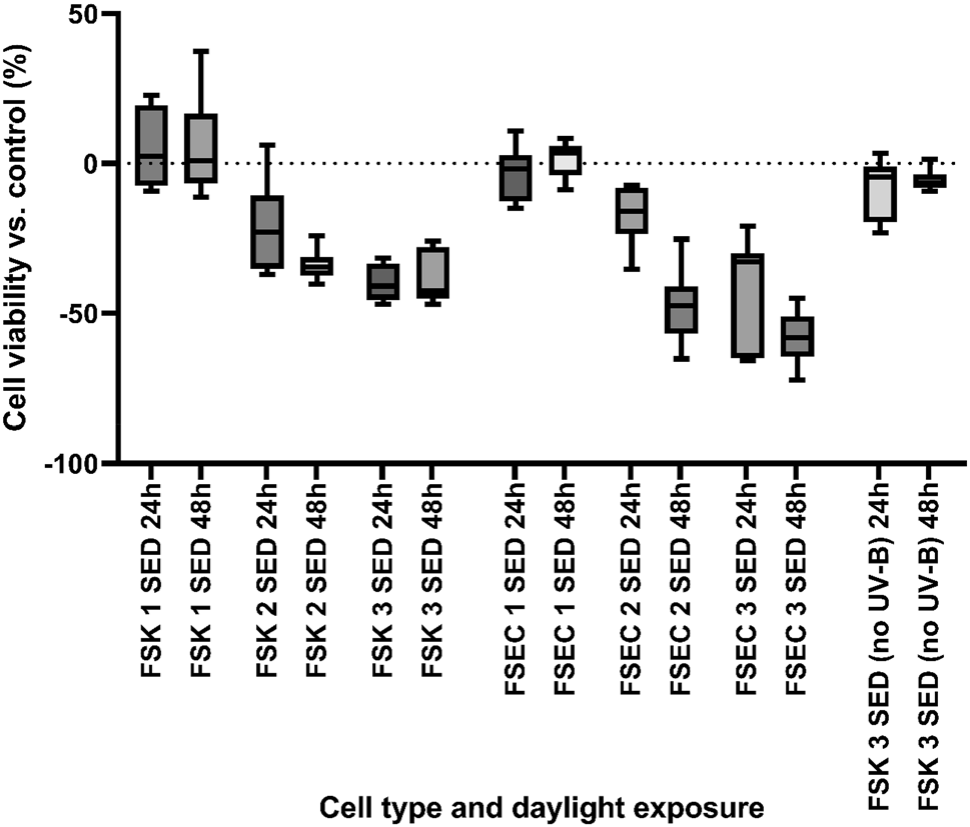
Three experiments carried out in triplicate using neonatal foreskin keratinocyte donor cell lines (FSK) and neonatal foreskin endothelial cell lines (FSEC). Cells were exposed to 1, 2 and 3 SED solar simulated sunlight and cell viability was recorded 24 and 48 h after exposure with use of the CCK-8 assay. Expressed as a percentage difference in cell viability vs. control sample. Boxplots display the median, 25th and 7th percentiles and whiskers display max and min values.

**Figure 4:**
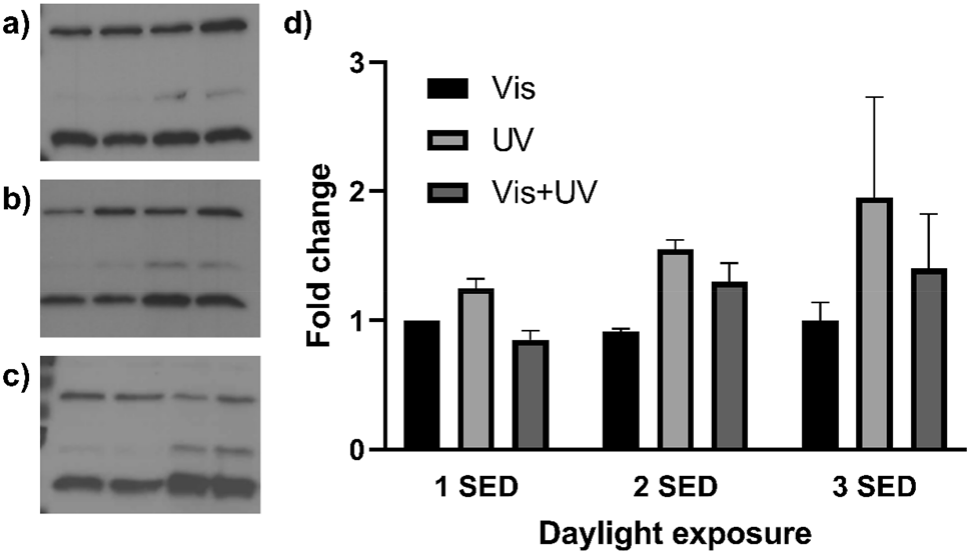
Western blotting of γ-H2AX in FSKs exposed to (**a**) 1 SED, (**b**) 2 SED or (**c**) 3 SED of sunlight (Full Western blot scans available in Supplementary Information S1). Exposures are quoted in the doses equivalent to full spectrum exposure and were carried out in summer months. (**d**) Expressed as fold change of γ-H2AX expression over control sample. Bars display geometric mean ± standard deviation.

**Figure 5:**
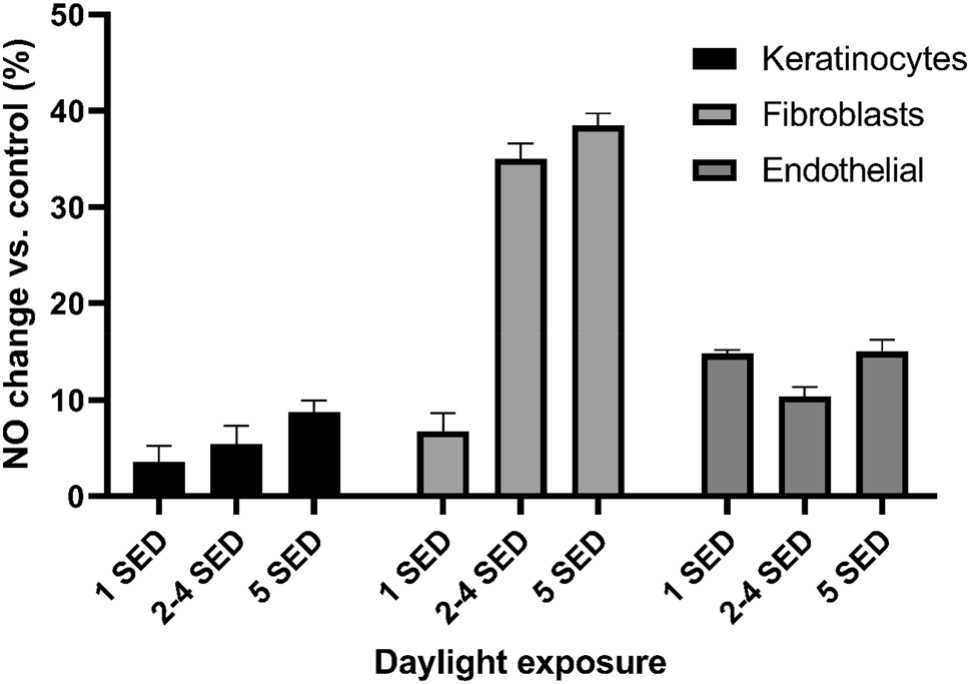
Nitric oxide production in keratinocyte, endothelial and fibroblasts skin cells following exposure to increasing doses of UK summers sunlight. Expressed as a percentage increase of NO expression vs. unirradiated control sample. Bars display geometric mean ± standard deviation.

**Figure 6:**
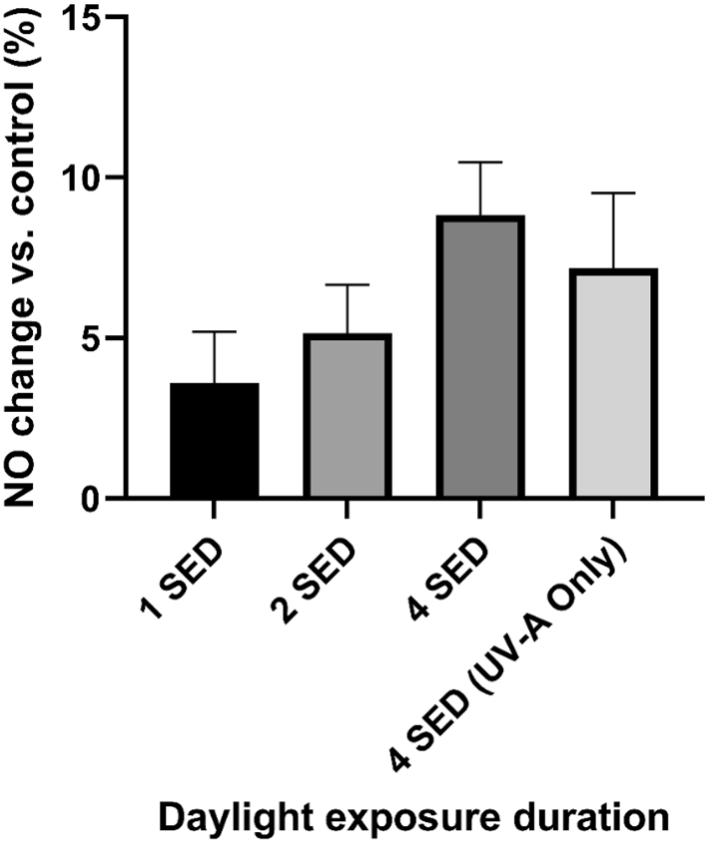
Dose response in keratinocytes at increased timescales of solar irradiance. Expressed percentage increase of NO expression vs. unexposed control. Bars display geometric mean ± standard deviation.

The fact that low dose sunlight exposure does not increase cell death and derives little elevation in DNA damage is particularly important if this level of exposure is sufficient to induce NO production (Figs. 3 and 4) through a UV-A route. In addition to FSK, we assessed this effect in FSEC and FSF skin cells situated deeper within the skin’s dermis. Even at the lowest doses (equivalent to 1 SED of full spectra irradiation) we found NO induction to occur in all skin cell types assayed; increasing doses of sunlight potentiated this effect, with FSK giving the lowest overall yield of NO, FSEC deriving the highest yields at low doses and FSF deriving highest yields at higher doses (Figs. 5 and 6). DNA damage and cell death assays suggested that adverse effects were directly proportional to increased sunlight exposures. This trend was also seen in nitric oxide induction for keratinocytes, although the trend was not significant in all cases (bar 15- and 30-min exposures) when each nearest higher exposure was considered.

### Shorter wavelength UV-A appears to be more efficient in sunlight-induced NO generation, from nitrite and the main mediator for effect seen, but whole UV-A spectrum is capable of NO generation

**Figure 7:**
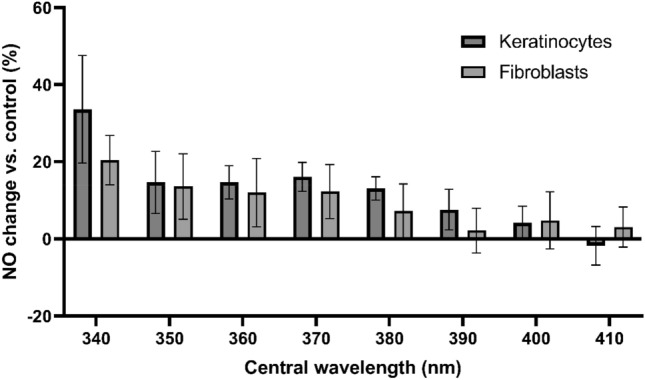
Production of nitric oxide from endothelial and keratinocyte skin cells after exposure to filtered simulated sunlight. Exposures were adjusted for filter transmission to produce 1.9 J/cm^2^. Expressed as percentage increase of NO expression vs. unexposed control. Bars display geometric mean ± standard deviation.

Initial results with UK sunlight exposure in an in vitro cell culture model suggested that the use of DAF-FM diacetate may be hindered by visible light. This was evident as UV-alone produced a marked upregulation of NO but when samples were exposed to full spectrum UK sunlight or visible light only, they appeared to produce less NO than the unexposed control (see supplementary information S1). Evaluation of this phenomenon suggested that DAF-FM DA photobleaching by visible light may be responsible for this effect. We hypothesised that this occurred due to the fact that excitation spectra of DAF-FM peaks at 495 nm, thereby full spectrum and visible light (> 420 nm) exposure induced photobleaching prior to quantitative analysis. To avoid this experimental artefact, a different compound, DAX-J2 red with excitation in the red spectral range, was used for experiments with simulated sunlight filtered by UV-A bandpass filters shown in Fig. 2. Use of DAX-J2 with FSK and FSEC cells suggested that the shorter UV-A wavelengths are considerably more efficient in NO production, consistent with results by others^15^; however, exposure to longer UV-A wavelengths also resulted in NO induction with lower efficacy (Fig. 7). This work matched previous findings suggesting that nitrite with a peak absorption in 340–360 nm is the main derivative broken down by UV-A in sunlight to NO^13,18,20,21^.

## Discussion

UV wavelengths within sunlight elicit DNA damage at different rates within skin cells, with UV-B up to 10,000-fold more damaging than longer UV-A^13^. In line with this thinking in vitro research demonstrates epidermal skin cells tolerate low level UV-A irradiation 7–9 J/cm^2^ (equivalent to ‘sub-erythemal’ doses of full spectrum sunlight) more effectively than UV-B^19^. Our work assessing exposures lower than 2 SED (that others claim do not provoke noticeable erythema in fair skinned Fitzpatrick type I individuals^22^ substantiate these claims, as in these instances 1 SED UK sunlight elicited little DNA damage in epidermal and dermal cell lines. However, at higher exposures (at or above 2 SED) a difference in cell survival was prevalent 48 h after exposure, with keratinocyte survival up to 50% higher compared with endothelial cell lines.

These results bring to the fore findings by D’Errico et al. and others who suggest skin cells tolerate sunlight exposure in a diverse manner, with ‘deeper’ dermal cells more susceptible to negative effects of sunlight than epidermal cells^23,24^. As keratinocytes form 90% of the skin’s epidermis providing the physical barrier between toxic insults within our environment, this line of thinking is indeed feasible^25^, however, as D’Errico’s work utilized fibroblasts as the dermal cell type, (suggesting a higher propensity for keratinocytes to remove UV induced signature mutations). Further work is needed to confirm if this effect transcends to endothelial cell lines and other dermal cell types.

Although our own in vitro work showed this effect differences in DNA damage tolerance between epidermal and dermal skin cells in vivo may in fact be much smaller through attenuation of short wavelength UV-B that is attenuated more effectively than other longer wavelengths^26^. Using this model and the UK summer solar spectrum, we estimated that 1 SED incident on the skin surface is attenuated to 0.0047 SED on the top of the dermis, to 0.0002 on the top of the basal layer and drops below 0.0001 in the dermis. Therefore, skin attenuation should indeed be taken into account when comparing damage tolerance of basal and dermal cells. Similarly, UV-A in sunlight is also attenuated by the skin, though to a lesser degree: to 0.069 when reaching the epidermis, 0.006–0.004 in the basal layer and drops below 0.0033 in the dermis. Our results therefore suggest longer wavelengths within the UV-A spectra reaching these deeper dermal cells could give rise to greater quantities of NO than in FSK (in which this trend is mostly associated with). This data backs up our earlier work which points out that UV-A can induce NO production in multiple skin cell lines for a considerable amount of time after exposure and is derived not only from the breakdown of salts^18^.

Although UV-B in sunlight is responsible for the majority of negative effects on the skin at high dose, these wavelengths constitute less than 5% of sunlight’s spectrum^1^, in comparison, visible and infra-red radiation is tenfold more predominant^1^. Contribution of these longer wavelengths in sunlight to skin damage was investigated in this study, either on their own or in conjunction with UV. No evidence of damage in skin cells by sunlight’s visible or infrared radiation at equivalent sub-erythemal and erythemal doses was found; instead, full spectrum sunlight exposure showed a trend towards lower fold changes in the DNA damage marker gamma H2AX in FSK compared with UV irradiation alone. This finding validates work by Barolet et al.^27,28^, who suggest that low levels of infra-red radiation within sunlight play a role in mitigating UV damage through DNA damage repair during the morning and evening when UV levels are low but infra-red and visible light remain high. It is therefore feasible that moderate sunlight exposure may promote protective responses in the skin.

In furthering these findings, it is worth considering evidence that NO is not simply a cardiovascular modulator but involved in upregulation of many inflammatory and immunological pathways which, in turn, touch a plethora of other organs through intermediates such as cyclooxygenase, prostaglandin pathways, cytokine’s and adipokines^29^. It is feasible then that in vivo low-level NO upregulation (as visualized here at 1 SED) may exert distinct effects on other organs. In vivo evidence highlights the plausibility of this suggesting that liver function, obesity, type 2 diabetes and metabolic syndrome may all be modulated via sunlight exposure independently of vitamin D synthesis^16,30^. This notion is clearly demonstrated in work by Dhamrait et al. suggesting that UV-A exposure affects weight gain and pro-diabetic effects indirectly through nitric oxide generation in obese mice on a high fat diet^31^. Others studies clarify this complex interplay giving evidence that NO like serotonin, melatonin and other mediators of the neuroendocrine axis is fundamental to induce effect in organs as far reaching as the brain, acting as a potent neurotransmitter, potentially permitting continuous cross talk between the skin and brain^32–36^, Hence the propensity of low level sunlight exposure to induce additional systemic biological effects alongside known cardiovascular benefits should not be discounted. Should this work proceed in vivo, this will permit the complex interplay between sunlight and its generated intermediates on human health to be better understood.

Finally, although use of UK sunlight for exposures may mitigate variability of results seen between some other studies due to use of different artificial light sources where spectral power distribution of irradiation varies, other caveats and limitations of this particular in-vitro set-up need to be considered. Firstly, the small number of samples doesn’t allow meaningful statistical analysis. This was, unfortunately, practically unavoidable as reproducible exposure of cell monolayers was only fulfilled under specific environmental conditions on non-cloudy midday around the summer solstice and clear of artifacts such as precipitation or fog. High variability of British weather limited opportunities for comparable experimental conditions for larger number of samples. In addition to this we also need to bear in mind other artifacts that are derived through use of cell culture monolayers such as the lack of repeat exposure—that others have suggested (from 7 to 9 J/cm^2^) when administered over an extended timeframe potentially derive adverse effects, not witnessed through ‘one-off’ irradiation^37^.

Alongside this is the Fitzpatrick scale (lost within an in-vitro model) may play a role in-vivo, potentially altering DNA damage long after the event via induction of ‘dark cyclobutene pyrimidine dimers (CPDs)’ and through the protective effect of melanin changing the threshold of direct DNA damage^38,39^ seen in our studies. Finally, lastly—and perhaps most importantly, is the complexity of the NO pathway in vivo with age, where reduced bioavailability of NO occurring through loss of function within the nitric oxide synthase enzyme (NOS) rather unfortunately gives rise to toxic reactive oxygen species superoxide and peroxynitrite, further compromising health status in these individuals. Hence, to truly feed into advice for healthy sunlight exposure we need to be mindful of these additional factors in vivo.

## To conclude

Our work suggests that epidermal and dermal skin cell lines produce the potent cardio-protectant NO under low dose UK sunlight (1 SED). Remarkably, this low-level exposure does not negatively affect cell survival and DNA damage witnessed at higher doses of sunlight, in line with others, it was shown that UV-A within the solar spectrum is the largest donor for NO induction, with a maximum of nitrite breakdown between 340 and 360 nm. However, it was also noted that NO generation was not restricted to this part of the solar spectrum and UVA was effective to generate NO with longer UV-A wavelengths near 400 nm. From a general health viewpoint, low dose sunlight may yield NO without significant adverse local effects on the skin, and it may aid some demographics whom in which NO has been categorically shown to decline with age.

### Supplementary Information

Supplementary Information.

## Data Availability

The datasets used and/or analyzed during the current study available from the corresponding author on reasonable request. All methods described above were performed in accordance with relevant guidelines and regulations.

## References

[1] Kricker A, Armstrong BK, English DR (1994). Sun exposure and skin cancer nonmelanocytic. Cancer Causes Control.

[2] Rittié L, Fisher GJ (2015). Natural and suninduced aging of human skin. Cold Spring Harb. Perspect. Med..

[3] Zigman S (1983). The role of sunlight in human cataract formation. Surv. Ophthalmol..

[4] Matsumura Y, Ananthaswamy HN (2004). Toxic effects of ultraviolet radiation on the skin. Toxicol. Appl. Pharmacol..

[5] Weller, R. B. Sunlight Has Cardiovascular Benefits Independently of Vitamin D. In: *Blood Purification*. S. Karger AG, pp.130–134. 10.1159/000441266 (2016). 10.1159/000441266 26766556

[6] Eppenberger LS, Sturm V (2020). The role of time exposed to outdoor light for myopia prevalence and progression: A literature review. Clin. Ophthalmol..

[7] Gorman S, Black LJ, Feelisch M, Hart PH, Weller R (2015). Can skin exposure to sunlight prevent liver inflammation?. Nutrients.

[8] Porojnicu AC, Robsahm TE, Dahlback A, Berg JP, Christiani D, Bruland ØS, Moan J (2007). Seasonal and geographical variations in lung cancer prognosis in Norway: Does Vitamin D from the sun play a role?. Lung Cancer.

[9] Wacker M, Holick MF (2013). Sunlight and Vitamin D: A global perspective for health. Dermatoendocrinol..

[10] Wright F, Weller RB (2015). Risks and benefits of UV radiation in older people: More of a friend than a foe?. Maturitas.

[11] Lingham G, MacKey DA, Lucas R, Yazar S (2020). How does spending time outdoors protect against myopia? A review. Br. J. Ophthalmol..

[12] Ikehata H, Ono T (2011). The mechanisms of UV mutagenesis. J. Radiat. Res..

[13] Holliman G, Lowe D, Cohen H, Felton S, Raj K (2017). Ultraviolet radiation induced production of nitric oxide: A multicell and multidonor analysis. Sci. Rep..

[14] Opländer C, Volkmar CM, PaunelGörgülü A, Faassen V, Heiss C, Kelm M, Halmer D, Mürtz M, Pallua N, Suschek CV (2009). Whole body UVA irradiation lowers systemic blood pressure by release of nitric oxide from intracutaneous photolabile nitric oxide derivates. Circ. Res..

[15] Pelegrino MT, Paganotti A, Seabra AB, Weller RB (2020). Photochemistry of nitric oxide and Snitrosothiols in human skin. Histochem. Cell Biol..

[16] Geldenhuys S, Hart PH, Endersby R, Jacoby P, Feelisch M, Weller RB, Matthews V, Gorman S (2014). Ultraviolet radiation suppresses obesity and symptoms of metabolic syndrome independently of vitamin D in mice fed a highfat diet. Diabetes.

[17] MalonePovolny, M. J., Maloney, S. E., & Schoenfisch, M. H. Nitric oxide therapy for diabetic wound healing. *Adv. Healthc. Mater.* **8**(12). 10.1002/adhm.201801210 (2019). 10.1002/adhm.201801210 PMC6774257 30645055

[18] Hazell G, Khazova M, Cohen H, Felton S, Raj K (2022). Postexposure persistence of nitric oxide upregulation in skin cells irradiated by UVA. Sci. Rep..

[19] Marionnet C, Tricaud C, Bernerd F (2015). Exposure to nonextreme solar UV daylight: Spectral characterization, effects on skin and photoprotection. Int. J. Mol. Sci..

[20] Liu D, Fernandez BO, Hamilton A, Lang NN, Gallagher JMC, Newby DE, Feelisch M, Weller RB (2014). UVA irradiation of human skin vasodilates arterial vasculature and lowers blood pressure independently of nitric oxide synthase. J. Investig. Dermatol..

[21] Opländer C, Suschek CV (2013). The role of photolabile dermal nitric oxide derivates in ultraviolet radiation (UVR) induced cell death. Int. J. Mol. Sci..

[22] Harrison, G.I., & Young, A.R. (n.d.). *Ultraviolet radiationinduced erythema in human skin*. [online] Available at: www.academicpress.com.

[23] D’errico, M., Teson, M., Calcagnile, A., Nardo, T., Luca, D., Lazzari, C., Soddu, S., Zambruno, G., Stefanini, M., & Dogliotti, E. (n.d.). *Differential Role of TranscriptionCoupled Repair in UVBInduced Response of Human Fibroblasts and Keratinocytes*. [online] Available at: www.aacrjournals.org. 15695384

[24] Goyeneche A (2020). Distinctive responses of keratinocytes and fibroblasts to sunlight-induced DNA damage. Invest. Ophthalmol. Vis. Sci..

[25] Nestle FO, Meglio D, Qin JZ, Nickoloff BJ (2009). Skin immune sentinels in health and disease. Nat. Rev. Immunol..

[26] Finlayson L, Barnard IRM, McMillan L, Ibbotson SH, Tom E (2022). Depth penetration of light into skin as a function of wavelength from 200 to 1000 nm. Photochem. Photobiol..

[27] Barolet D (2021). Near-infrared light and skin: ‘Why intensity matters. Curr. Probl. Dermatol..

[28] Barolet D, Christiaens F, Hamblin MR (2016). Infrared and skin: Friend or foe. J. Photochem. Photobiol. B Biol..

[29] Man MQ, Wakefield JS, Mauro TM, Elias PM (2022). Regulatory role of nitric oxide in cutaneous inflammation. Inflammation.

[30] Hart PH, Gorman S, Finlay-Jones JJ (2011). Modulation of the immune system by UV radiation: More than just the effects of vitamin D?. Nat. Rev. Immunol..

[31] Dhamrait GK, Panchal K, Fleury NJ, Abel TN, Ancliffe MK, Crew RC, Croft K, Fernandez BO, Minnion M, Hart PH, Lucas RM, Mark PJ, Feelisch M, Weller RB, Matthews V, Gorman S (2020). Characterising nitric oxide-mediated metabolic benefits of low-dose ultraviolet radiation in the mouse: A focus on brown adipose tissue. Diabetologia.

[32] Kanuri BN, Kanshana JS, Rebello SC, Pathak P, Gupta AP, Gayen JR, Jagavelu K, Dikshit M (2017). Altered glucose and lipid homeostasis in liver and adipose tissue predispose inducible NOS knockout mice to insulin resistance. Sci. Rep..

[33] Martín MJ, Jiménez MD, Motilva V (2001). New issues about nitric oxide and its effects on the gastrointestinal tract. Curr. Pharmaceut. Des..

[34] Nelson, R.J., Kriegsfeld, L.J., Dawson, V.L., & Dawson, T.M. *Effects of nitric oxide on neuroendocrine function and behavior* (1997). 10.1006/frne.1997.0156 9344634

[35] Slominski AT, Zmijewski MA, Plonka PM, Szaflarski JP, Paus R (2018). How UV light touches the brain and endocrine system through skin, and why. Endocrinology.

[36] Slominski AT, Slominski RM, Raman C, Chen JY, Athar M, Elmets C (2022). Neuroendocrine signaling in the skin with a special focus on the epidermal neuropeptides. Am. J. Physiol. Cell Physiol..

[37] Seité S, Fourtanier A, Moyal D (2010). Photodamage to human skin by suberythemal exposure to solar ultraviolet radiation can be attenuated by sunscreens: A review. Br. J. Dermatol..

[38] Fajuyigbe D, Douki T (2021). Dark cyclobutane pyrimidine dimers are formed in the epidermis of Fitzpatrick skin types I/II and VI in vivo after exposure to solarsimulated radiation. Pigment Cell Melanoma Res..

[39] Supp DM, Hahn JM, Lloyd CM, Combs KA, Swope VB, AbdelMalek Z, Boyce ST (2020). Light or dark pigmentation of engineered skin substitutes containing melanocytes protects against ultraviolet lightinduced DNA damage in vivo. J. Burn Care Res..

