# Supplementary material for: Low-dose daylight exposure induces nitric oxide release and maintains cell viability in vitro
Source: Sci Rep. 2023 Sep 28;13:16306. doi: 10.1038/s41598-023-43653-2 (PMC10539323; doi:10.1038/s41598-023-43653-2)
Supplement: Supplementary file 1 — Supplementary Information. [file 41598_2023_43653_MOESM1_ESM.docx]

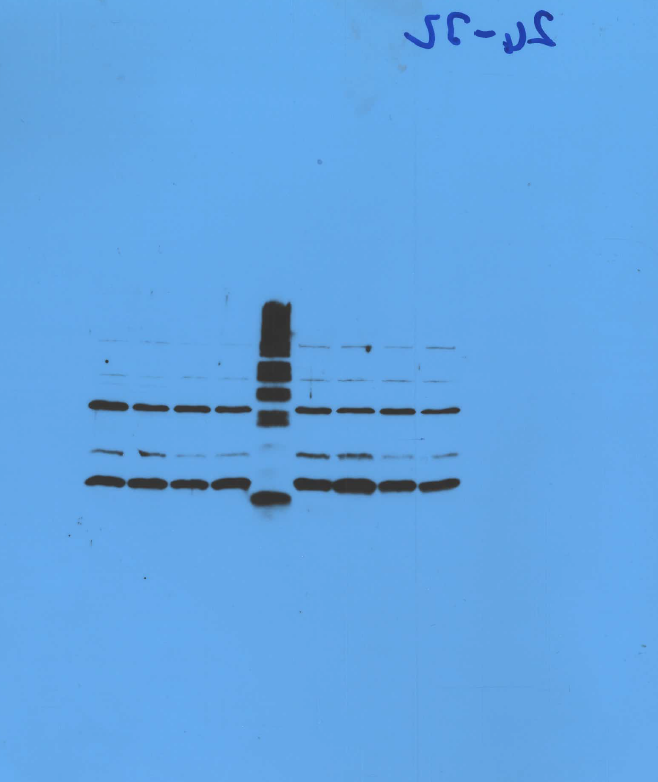
1 SED sunlight exposure Keratinocytes – used for figure 5.

| Lane | Sample | Dose of sunlight |
| --- | --- | --- |
| 1 | Unexposed donor 1 | 1 SED |
| 2 | Visible light treated donor 1 | 1 SED |
| 3 | UV only exposed donor 1 | 1 SED |
| 4 | Full spectrum sunlight donor 1 | 1 SED |
| 5 | Ladder |  |
| 6 | Unexposed donor 2 | 1 SED |
| 7 | Visible light treated donor 2 | 1 SED |
| 8 | UV only exposed donor 2 | 1 SED |
| 9 | Full spectrum sunlight donor 2 | 1 SED |

Phospho-H2AX expression at 1 SED sunlight exposure for 2 neonatal donor cell lines (keratinocytes).

2 SED sunlight exposure Keratinocytes – used for figure 5.

| Lane | Sample | Dose of sunlight |
| --- | --- | --- |
| 1 | Ladder |  |
| 2 | Unexposed donor 1 | 2 SED |
| 3 | Visible light treated donor 1 | 2 SED |
| 4 | UV only exposed donor 1 | 2 SED |
| 5 | Full spectrum sunlight donor 1 | 2 SED |
| 6 | Unexposed donor 2 | 2 SED |
| 7 | Visible light treated donor 2 | 2 SED |
| 8 | UV only exposed donor 2 | 2 SED |
| 9 | Full spectrum sunlight donor 2 | 2 SED |


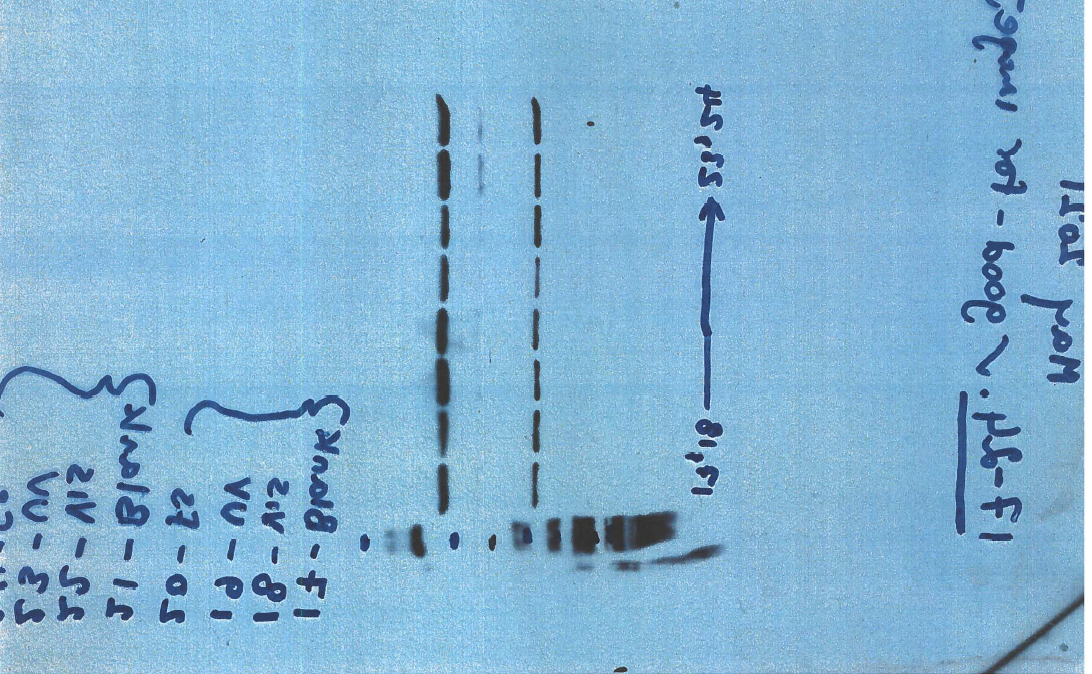


Phospho-H2AX expression at 2 SED sunlight exposure for 2 neonatal donor cell lines (keratinocytes).

3 SED sunlight exposure Keratinocytes – used for figure 5.


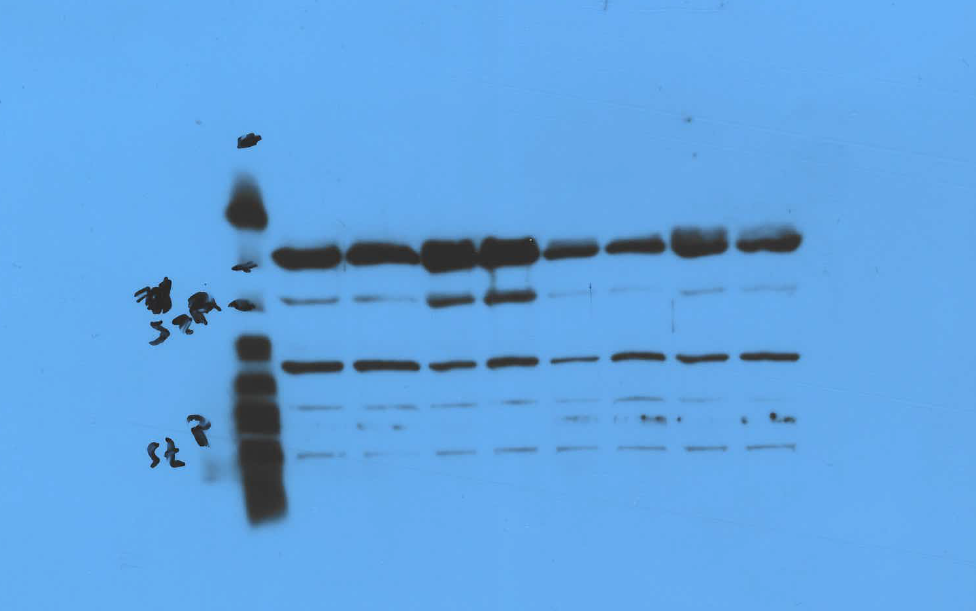


| Lane | Sample | Dose of sunlight |
| --- | --- | --- |
| 1 | Ladder |  |
| 2 | Unexposed donor 1 | 3 SED |
| 3 | Visible light treated donor 1 | 3 SED |
| 4 | UV only exposed donor 1 | 3 SED |
| 5 | Full spectrum sunlight donor 1 | 3 SED |
| 6 | Unexposed donor 2 | 3 SED |
| 7 | Visible light treated donor 2 | 3 SED |
| 8 | UV only exposed donor 2 | 3 SED |
| 9 | Full spectrum sunlight donor 2 | 3 SED |

B.

Phospho-H2AX expression at 3 SED sunlight exposure for 2 neonatal donor cell lines (keratinocytes).
